# Hearing rehabilitation for unilateral deafness using a cochlear implant: the influence of the subjective duration of deafness on speech intelligibility

**DOI:** 10.1007/s00405-022-07531-3

**Published:** 2022-07-06

**Authors:** Tobias Rader, Oliver Julian Waleka, Sebastian Strieth, Klaus Wolfgang Georg Eichhorn, Andrea Bohnert, Dimitrios Koutsimpelas, Christoph Matthias, Benjamin Philipp Ernst

**Affiliations:** 1 Division of Audiology, Department of Otorhinolaryngology, Ludwig-Maximilians-University Medical Center, Munich, Germany; 2 Department of Otorhinolaryngology, University Medical Center Mainz, Mainz, Germany; 3 Department of Otorhinolaryngology, University Medical Center Bonn (UKB), Bonn, Germany; 4 LMU Klinikum, Klinik für Hals-Nasen-Ohrenheilkunde, Abteilung Audiologie, Marchioninistr. 15, 81377 Munich, Germany

**Keywords:** Hearing rehabilitation, Single sided deafness, Cochlear implant, Duration of deafness, Questionnaire

## Abstract

**Background:**

For patients with single sided deafness (SSD) or severe asymmetric sensorineural hearing loss (ASHL), cochlear implantation remains the only solution to restore bilateral hearing capacity. Prognostically, the duration of hearing loss in terms of audiological outcome is not yet clear. Therefore, the aim of this study was to retrospectively investigate the influence of subjective deafness duration on postoperative speech perception after cochlear implantation for SSD as well as its impact on quality of life.

**Materials and methods:**

The present study included a total of 36 adults aged 50.2 ± 15.5 years who underwent CI for SSD/ASHL at our clinic between 2010 and 2015. Patients were audiometrically assessed at 3 and 12–36 months postoperatively. Test results were correlated with self-reported duration of deafness. Quality of life was assessed by questionnaire.

**Results:**

Mean duration of deafness was 193.9 ± 185.7 months. The side-separated hearing threshold showed an averaged target range between 30 and 40 dB HL. Freiburg monosyllable test increased from 0% pre-operatively to 20% after 3 months (*p* = 0.001) and to 50% after 12–36 months (*p* = 0.002). There was a significant correlation between audiometric outcome and subjective deafness duration at 12–36 months postoperatively (*r* = − 0.564; *p* = 0.02) with a cutoff for open-set monosyllable recognition at a duration of deafness of greater than 408 months. Quality of life was significantly improved by CI.

**Conclusions:**

CI implantation in unilaterally deafened patients provides objective and subjective benefits. Duration of deafness is unlikely to be an independent negative predictive factor and thus should not generally be considered as contraindication.

## Introduction

Patients with single sided deafness (SSD) and a normal-hearing contralateral ear are severely limited in various aspects of their daily lives [1]. These limitations due to monaural hearing are particularly evident in directional hearing (localization of sound sources), speech understanding in noise, and auditory effort [2–4].

In acquired single sided deafness (SSD), a cochlear implant (CI) is the best possible treatment option to largely restore binaural hearing in affected patients. CI provision is also widely established in congenital born unilaterally deaf children [5–9]. In addition to the positive effects of the CI on speech comprehension in noise, localization of sound sources and reduced hearing effort, affected patients also show a significant reduction of tinnitus [10–13].

An influence of the normal-hearing opposite ear as an influence of central speech processing in SSD patients is shown by significantly worse hearing in the better ear compared to the age-correlated normal-hearing (NH) comparison group [14].

Predictive factors for hearing rehabilitation success via cochlear implantation are widely reported. Duration of deafness is a recognized factor in predicting hearing success [15–17]. This paper retrospectively investigates the influence of self-reported duration of deafness on postoperative speech perception after cochlear implantation for SSD as well as its impact on quality of life.

## Materials and methods

In the present study, hearing rehabilitation in SSD by means of CI fitting is further investigated within the framework of a retrospective data analysis. For this purpose, 36 patients were identified who were fitted with a CI for SSD at the Department of Otorhinolaryngology of the University Medical Center of the Johannes Gutenberg University Mainz between 2010 and 2015.

Patient specific data with regard to date of birth, gender, self-reported duration of deafness, side of deafness, cause of deafness, history of hearing aid/CROS fitting, history of tinnitus complaints, date of surgery, implant and electrode type, as well as audiometric data and subjective patient satisfaction were recorded.

Follow-up audiometric data were collected and analyzed at 3 months and at 12–36 months after surgery. Quality of life was assessed by questionnaire.

### Audiological examinations

All patients who were evaluated underwent comprehensive audiological examinations as part of the preliminary examinations for cochlear implantation. The examinations were performed in soundproof booths with calibrated audiometers.

Pure-tone audiometry for air- and bone-conduction was tested separately for both ears at the following frequencies: 125, 250, 500, 1000, 2000, 3000, 4000, 6000, and 8000. Masking of the better hearing ear was performed to rule out cross hearing using narrowband noise. If no hearing threshold could be recorded due to deafness, the hearing threshold at this frequency was set to the value of 130 dB to perform a statistical evaluation of the hearing threshold.

For the evaluation of speech intelligibility in quiet, the Freiburg speech intelligibility test (using monosyllables and numbers) according to Hahlbrock [18–20] was used. Testing was performed preoperatively using headphones and postoperatively in the open field at a speech presentation level of 65 dB SPL. The non-implanted contralateral ear was masked during the measurement in the free field via insert earphones with CCITT noise $\ge$ 65 dB.

### Quality of life questionnaires

Quality of life was assessed with the Bern Benefit *in Single Sided* Deafness Questionnaire (BBSS) [21]. The test consists of 10 items, each divided into a scale from − 5 ("much easier without device") to 5 ("much easier with device"). The respondent can enter the level that applies to him or her, analogous to a numerical pain scale. The questionnaire measures subjective patient satisfaction and compares it to the state of hearing care before cochlear implantation. The BBSS questionnaire was used to assess the pre- and post-operative status in a single questionnaire.

### Statistical evaluation

The statistical analysis of the results and the comparison of the individual groups with each other were carried out using non-parametric rank tests with the aid of SPSS software (IBM/NY, USA). Here, *p*-values < 0.05 were considered significant and < 0.01 as highly significant. The Wilcoxon signed rank test was used for intergroup comparison, and the one-sample signed rank test was used for the intragroup test. Correlation was calculated according to Pearson (two-tailed). Results are presented as median or rounded mean values with the respective standard deviation (± SD), unless otherwise indicated.

## Results

### Patient collective

In the present study, data of 36 patients (22 female, 14 male) were retrospectively collected and analyzed who were fitted with a CI due to acquired SSD or asymmetric sensorineural hearing loss. All patients were fitted with CROS- or high power hearing aids depending upon residual hearing capacities. The mean age at implantation was 50.2 ± 15.5 years. 22 (61.1%) patients received left-sided and 14 (38.9%) right-sided CIs.

22 (61.1%) of the patients were implanted with a MED-EL CI system from MED-EL and 14 (38.9%) with a cochlear CI system. The tabular listing of the electrode array used in combination with the implant in each case and the corresponding frequency distribution is shown in Table 1.

**Table 1:** Number and frequency distribution of cochlear implant models and associated electrode types

Manufacturer	Model	Electrode type	Implantation	
			Quantity	Percent (%)
MED-EL	SYNCHRONY	FLEX^28^	8	22.2
	CONCERTO	FLEX^soft^	1	2.8
		FLEX^28^	10	27.8
		FLEX ^24^	1	2.8
		Compressed	2	5.6
Cochlear	CI522	Slim straight	2	5.6
	CI512	Contour advance	5	13.9
	CI422	Slim straight	7	19.4

### Causes of deafness

The mean self-reported duration of deafness of the patients before cochlear implantation was 193.9 ± 185.7 months (min: 1; max: 660 months). The following causes for deafness were reported: Hearing loss (58.3%), congenital progressive hearing loss (11.1%), acoustic neuroma (8.3%), tympanoplasty (8.3%), and temporal bone fracture (5.6%).

### Tinnitus

More than two thirds of the patients (66.7%) complained of tinnitus symptoms in addition to unilateral hearing loss. One third (33.3%) of the patients described a tinnitus ipsilateral to the deafness, 27.8% a bilateral tinnitus and 5.6% a contralateral manifestation.

### Pure tone audiometry

**Fig. 1:**
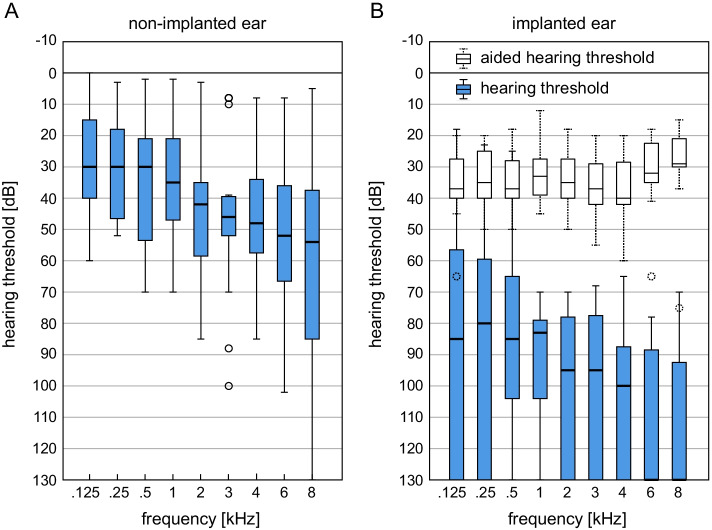
Boxplot representation of the preoperatively determined pure tone audiometry hearing thresholds (*n* = 36) in the **A** non-implanted and **B** later implanted ear. In addition, the aided long-term hearing thresholds (12–36 months) measured in free field condition with CI are shown. Boxplot representation with median, quartiles and maximum values. Circles represent outliers

Figure 1 shows the hearing thresholds for the non-implanted and the implanted ear determined preoperatively by pure tone audiometry using headphones. The figure shows that these are patients with unilateral normal hearing or conventionally with hearing aids treatable low-grade hearing *loss* (*asymmetric sensorineural hearing loss,* ASHL). The median hearing loss across all 9 measured test frequencies is 39.0 dB HL in the non-implanted ear and 100.5 dB HL in the implanted ear.

### Speech audiometry

**Fig. 2:**
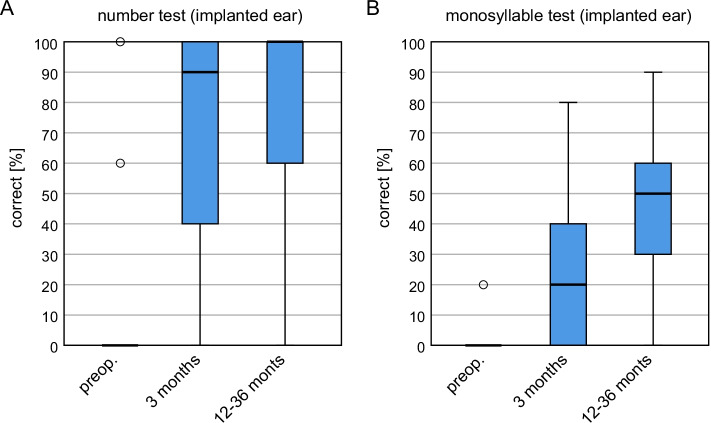
Boxplot representation of the **A** Freiburg number and **B** Freiburg monosyllable test score at the implanted ear over time at the time points preoperatively, 3 months and 12–36 months after implantation. Boxplot representation with median, quartiles and maximum values. Circles represent outliers

Figure 2 shows the results of the Freiburg Number and Monosyllable Test at the time points preoperatively, 3 and 12–36 months, postoperatively. Preoperatively, the test was performed via headphones, at the postoperative time points in the open field with active masking of the non-implanted ear.

The result in the Freiburg number test shows a significant increase from a median of 0% to 90% after 3 months (*p* < 0.001) and from 90 to 100% in the long-term course (*p* > 0.05). The result in the Freiburg monosyllable test increases from 0% pre-operatively to 20% after 3 months (*p* = 0.001) and from 20 to 50% in the long-term course (*p* = 0.002). 50% of the patients show a percentage of monosyllable comprehension in the long-term course between 30 and 60%.

### Relationship between duration of deafness and speech comprehension

**Fig. 3:**
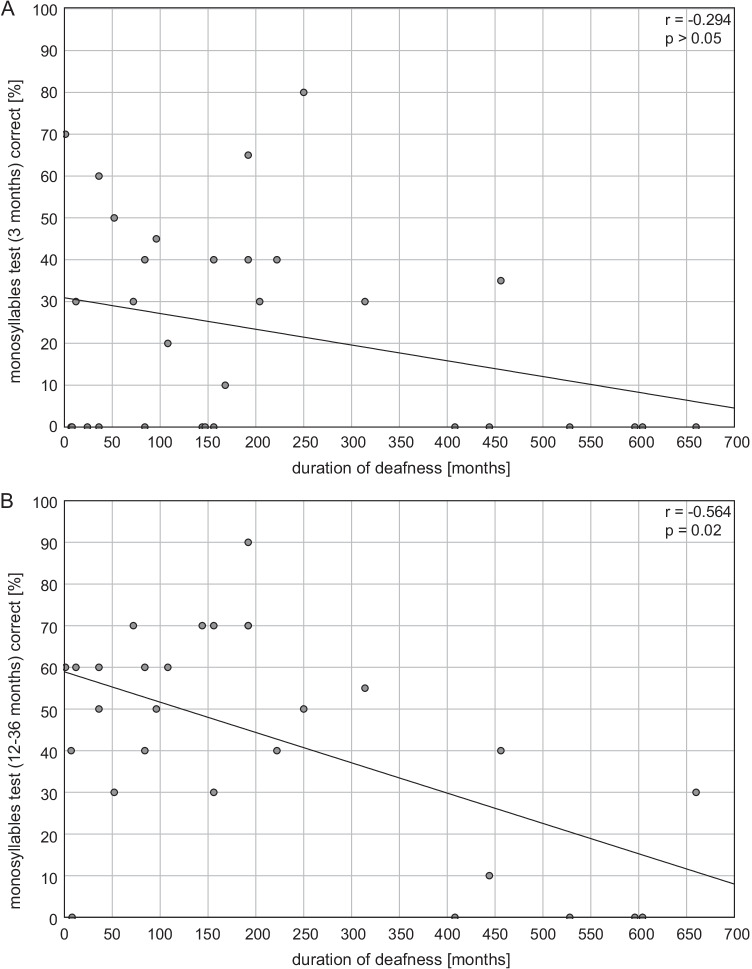
Correlation between subjective deafness duration and monosyllable understanding of the implanted ear for **A** 3 months and **B** 12–36 months after implantation

If the duration of deafness is compared with the speech understanding at the time points 3 months and 12–36 months as a correlation, a non-significant correlation according to Pearson (*r* = − 0.294; *p* > 0.05) is shown for the time point 3 months and a highly significant correlation (*r* = − 0.564; *p* = 0.02) for the time point 12–36 months (Fig. 3). At 12–36 months after implantation, all patients (except one) who do not show open-set monosyllable recognition after this time point have a duration of deafness greater than 408 months (34 years). In 6 of 7 patients with a duration of deafness < 156 months (13 years) who did not show open-set monosyllable recognition at 3 months achieved a score of ≥ 30% at 12–36 months.

### BBSS quality of life questionnaire

**Fig. 4:**
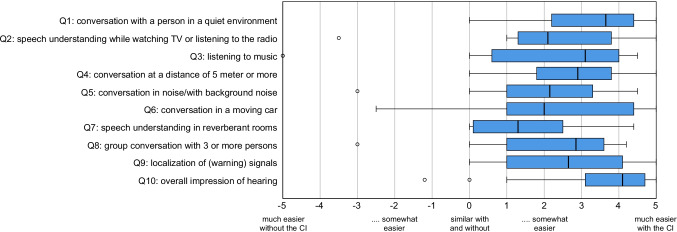
Results of the BBSS questionnaire. Q1–Q10 indicates question number. Boxplot representation with median, quartiles, and maximum values. Circles represent outliers

Figure 4 shows the results of the BBSS questionnaire. The individual answers of the patients are shown as boxplots within the proficiencies from − 5 (much easier without CI) to 5 (much easier with CI). The medians, like the markers of the 25% quartiles, are also in the positive range in all 10 items, i.e., better with CI device. The lowest median value was found in question 7 for the item "speech understanding in reverberant rooms" with 1.3. For question 10 with the item "overall impression of hearing" the highest satisfaction was found with CI with a value of 4.1. From the sub-questions in the BBSS the average value over all questions is calculated as "total score" with a value of 2.4.

## Discussion

The present retrospective evaluations show that unilaterally deaf patients achieve satisfactory speech understanding in addition to strong subjective satisfaction. The aim of this study was to analyze whether the duration of deafness before implantation in these CI users is a negative predictive factor for their postoperative *outcome*.

### Speech audiometry

In Freiburg monosyllable comprehension test, patients show a median increase over the preoperative measurement (0%) to 20% in the first three months and to 50% for the period 12–36 months. The data of this evaluation show a slightly more favorable speech understanding than the data published by Sullivan et al. using the consonant-nucleus-consonant (CNC) speech test with results of 32% and 30% for the time points 3 and 6 months after implantation to about 35% for the period 12–36 months. [13]. On average, these results measured with CI alone and masked counter ear are worse compared to "classic" CI patients without SSD. For example, data from Hoppe on 284 cases 6 months after implantation show a monosyllable understanding of monosyllables between 65 and 85%. [22]. This is due to the fact that in SSD the normal hearing ear takes over the main task of speech discrimination and supports the CI ear, especially in difficult noise situations and directional hearing. [23].

It should be emphasized that the significant improvement in speech understanding between the periods 3 months postoperatively and 12–36 months postoperatively shown in the present collective is accompanied by no change in the hearing threshold in the inflation curve. This is most likely to be explained by training effects of the parallel audioverbal therapy or by an improvement of central auditory processing, respectively, and is in accordance with the literature [24, 25].

### Influence of the duration of deafness on speech understanding

The main question of this work was the relationship between self-reported duration of deafness and postoperative monosyllabic comprehension.

First, the result after 3 months postoperatively was considered as an approximation. Here, the analysis of the linear regression according to Pearson showed a tendency towards a negative correlation between self-reported duration of deafness and postoperative outcome in the Freiburg monosyllable comprehension, however, without reaching significance level (*r* = − 0.294; *p* > 0.05). In accordance with the a results of other study groups, no correlation between deafness duration and speech understanding can be identified in the present collective at this time [26].

Looking at the results of the linear regression analysis 12–36 months after CI fitting, there is a clear and highly significant correlation between self-reported duration of deafness and speech understanding after CI fitting (*r* = − 0.564; *p* < 0.01). This result is supported by the individual analysis of the single cases. At 3 months postoperatively, only one out of seven patients (14.3%) with a self-reported duration of deafness greater than 400 months yielded a monosyllabic understanding of ≥ 30%. At 12–36 months, this ratio increased to two out of seven patients (28.6%) while one patient was able to achieve a monosyllabic understanding of 10%. Consequently, four out of seven patients (57.1%) with a self-reported duration of deafness greater than 400 months showed no monosyllabic understanding. However, for deafness durations of less than 400 months, 28 of 29 patients (96.6%) were able to achieve a monosyllabic understanding of ≥ 30%. In case of a self-reported duration of deafness of less than 156 months, a majority of patients (85.7%) who had no open monosyllabic understanding at 3 months showed monosyllabic understanding of ≥ 30% at 12–36 months. Accordingly, the prognosis for successful hearing rehabilitation with CI in our collective can be assumed to be favorable for deafness durations of less than 400 months. Successful hearing rehabilitation with CI for longer deafness durations must be regarded as clearly limited but nevertheless feasible. In line with this, Nassiri et al. could not find any difference in the hearing success of SSD patients with CI between a deafness duration of more or less than 10 years [26].

Furthermore, it is unclear whether the duration of deafness alone or whether other factors, such as age-related factors, play a role in determining the prognosis of patients with deafness durations exceeding 33 years. The etiology of the hearing impairment is of central importance [27]. The majority of patients (77.7%) in the present collective developed SSD as a result of a sudden hearing loss, congenital progressive hearing loss, or after tympanoplasty. These etiologies represent typical causes of cochlear hearing loss without evidence of a neural component. This contrasts with patients who developed SSD as a result of resection of a vestibular schwannoma (8.3%) or after temporal bone fracture (5.6%). Hearing loss of these etiologies may occur in combination with neural or central hearing loss in addition to cochlear hearing loss [28, 29]. This, in turn, may negatively affect the prognosis of hearing gain with CI or the results of the present collective.

Other possible influences are, for example, the use of a hearing aid before CI fitting, the implantation site, the absence of cardiovascular risk factors, and the residual hearing in the fitted ear [30]. Of course, the quality of surgical care (surgeon experience, clinic caseload, etc.) should also be considered, as technical factors also influence outcome [31].

In addition to the pure prognosis in terms of hearing rehabilitation, especially in elderly patients, the proven positive impact on cognitive function should also be considered [32, 33]. Since hearing loss represents an independent and treatable risk factor for the development of dementia, this should be discussed in detail during patient counseling and evaluated as a therapeutic rationale in light of the limited prognosis.

Patients with above-average deafness duration represent a phenomenon CI clinics in general were confronted with after the establishment of the indication for CI in SSD patients in 2008 [11]. Since only conservative hearing aid fittings using conventional hearing aids, *Contralateral Routing of Signals* (CROS) or BiCROS hearing aids, or implantable bone conduction hearing systems were available for these patients until then, the newly established indication resulted in a large collective of long-term deafened patients for whom the option of CI was available for the first time. Accordingly, the number of patients with long-term deafness presenting for CI evaluation could potentially be expected to decline in the coming decades against the background of widespread availability of CI fitting.

### Quality of life assessment

The study of quality of life represents an important endpoint for a health and medical research [34]. Patients assessed their respective hearing impression in certain situations using a predefined scale.

The patients with SSD rated their hearing impression with CI better than compared to their condition preoperatively without CI. Thus, the median values of all questioned items are shifted to the right on the scale, which reflects a more positive hearing impression with hearing aid. Overall, the answers are relatively homogeneous with interquartile range values also in the positive range (range between 25 and 75%).

Several studies show that hearing via CI can result in limited music enjoyment due to the technical limitations of a CI [35, 36]. The good rating of the item music in the BBSS can be justified by the special patient population of SSD, who in the "*best aided condition*" with CI and normal hearing on the opposite side, certainly absorb music more with the normal hearing ear.

Question 7 "Conversation in reverberant rooms" is rated as the most difficult situation with a CI at the median in the BBSS. Conversation in reverberant rooms is also rated as a difficult listening situation in other studies [37, 38].

The result of question 10 with the item "Overall impression of hearing" is gratifying. The improvement shown here for unilaterally deafened patients after implantation with a CI is also evident in other studies such as Jacob et al. and Prejban et al. [39, 40]. For example, Jacob et al. showed an acceptance of CI- care for 13 patients with SSD of 100%. Prejban et al. describe a benefit after CI fitting especially in difficult listening situations. Both studies postulate that the restoration of hearing by means of CI is also a reasonable therapy option for SSD.

### Limitations of the study

Limitations of the present study are primarily due to the study design. This was a retrospective patient population from a single CI center. Due to the retrospective study design, audiometric control was not available for all patients at uniform time points. This resulted in the comparison three months postoperatively, which was available for all patients, and 12–36 months postoperatively. In addition, Oldenburg sentence test results were available for only a small proportion of patients, so that only the Freiburg sentence tests available for all patients were evaluated. Consequently, no statements can be made about the significantly more demanding hearing in noise.

Patients with SSD and ASHL of different etiologies were also included. Accordingly, it is not possible to differentiate between the influence of SSD or ASHL on hearing success with CI. In addition, a considerable proportion of patients had unusually long subjective deafness durations due to the newly established indication for CI in SSD. Furthermore, etiologies of hearing impairment were present, which in addition to cochlear hearing loss may also cause neural or central hearing impairment. In conclusion, the BBBS questionnaire used to assess quality of life after unilateral CI fitting is basically developed for assessment after fitting using *Bone Anchored Hearing Aid* (BAHA).

## Summary

Overall, this work showed that CI implantation in unilaterally deafened patients provides objective and subjective benefits. The current research findings support these results and show that the use of a CI in SSD will continue to increase in the future.

Based on these findings, the subjective duration of deafness before implantation in the deafened ear alone should not be the sole contraindication against CI in SSD. It is still not clear which preoperative factors are a precise predictor for the postoperative speech understanding in individual cases.

In perspective, the targeted use of patient-specific anatomical (e.g., the insertion angle of the CI electrodes) and physiological data (e.g., the pitch mapping of individual CI electrodes) and their inclusion in speech processing strategies will also lead to a better more realistic mapping of the acoustic situation during electrical stimulation via the CI [41, 42].

## References

[1] Wie OB, Pripp AH, Tvete O (2010). Unilateral deafness in adults: effects on communication and social interaction. Ann Otol Rhinol Laryngol.

[2] Döge J, Baumann U, Weissgerber T (2017). Single-sided deafness//Single-sided deafness: impact of cochlear implantation on speech perception in complex noise and on auditory localization accuracy: impact of cochlear implantation on speech perception in complex noise and on auditory localization accuracy. Otol Neurotol.

[3] Hoth S, Rosli-Khabas M, Herisanu I (2016). Cochlear implantation in recipients with single-sided deafness: audiological performance. Cochlear Implants Int.

[4] Arndt S, Laszig R, Aschendorff A (2016). Cochleaimplantatversorgung bei einseitiger Taubheit oder asymmetrischem Horverlust // Cochleaimplantatversorgung bei einseitiger Taubheit oder asymmetrischem Hörverlust (Cochlear implant treatment of patients with single-sided deafness or asymmetric hearing loss. German version). HNO.

[5] Deep NL, Gordon SA, Shapiro WH (2020). Cochlear Implantation in Children with Single-Sided Deafness. Laryngoscope.

[6] Ehrmann-Mueller D, Kurz A, Kuehn H (2020). Usefulness of cochlear implantation in children with single sided deafness. Int J Pediatr Otorhinolaryngol.

[7] Purcell PL, Cushing SL, Papsin BC (2020). Unilateral hearing loss and single-sided deafness in children: an update on diagnosis and management. Curr Otorhinolaryngol Rep.

[8] Rauch A-K, Arndt S, Aschendorff A (2020). Long-term results of cochlear implantation in children with congenital single-sided deafness. Eur Arch Otorhinolaryngol.

[9] Ramos Macías Á, Borkoski-Barreiro SA, Falcón González JC (2019). Single-sided deafness and cochlear implantation in congenital and acquired hearing loss in children. Clin Otolaryngol.

[10] Buechner A, Brendel M, Lesinski-Schiedat A (2010). Cochlear implantation in unilateral deaf subjects associated with ipsilateral tinnitus. Otol Neurotol.

[11] De Heyning PV, Vermeire K, Diebl M (2008). Incapacitating unilateral tinnitus in single-sided deafness treated by cochlear implantation. Ann Oto Rhinol Laryn.

[12] Speck I, Challier P, Wesarg T (2020). Is the cochlear implant a successful long-term solution for single-sided deaf and asymmetric hearing-impaired patients?. Eur Arch Otorhinolaryngol.

[13] Sullivan CB, Al-Qurayshi Z, Zhu V (2020). Long-term audiologic outcomes after cochlear implantation for single-sided deafness. Laryngoscope.

[14] Arndt S, Wesarg T, Stelzig Y (2019). Einfluss einseitiger Taubheit auf das Hörvermögen des besseren Ohrs (Influence of single-sided deafness on the auditory capacity of the better ear. German version). HNO.

[15] Kitterick PT, Lucas L (2016). Predicting speech perception outcomes following cochlear implantation in adults with unilateral deafness or highly asymmetric hearing loss. Cochlear Implants Int.

[16] Firszt JB, Reeder RM, Holden LK (2018). Results in adult cochlear implant recipients with varied asymmetric hearing: a prospective longitudinal study of speech recognition, localization, and participant report. Ear Hear.

[17] Cohen SM, Svirsky MA (2019). Duration of unilateral auditory deprivation is associated with reduced speech perception after cochlear implantation: a single-sided deafness study. Cochlear Implants Int.

[18] Hahlbrock K-H (1953). Über Sprachaudiometrie und neue Wörterteste (Speech audiometry and new word tests). Arch Ohren Nasen Kehlkopfheilkd.

[19] DIN (1995) Tonträger mit Sprache für Gehörprüfung - Teil 1: Tonträger mit Wörtern nach DIN 45621–1 (Aufnahme 1969)(DIN 45626–1:1995–08)

[20] DIN EN ISO 8253–3: Akustik - Audiometrische Prüfverfahren - Teil 3: Sprachaudiometrie (ISO 8253–3:2012); Deutsche Fassung EN ISO 8253–3:2012

[21] Kompis M, Pfiffner F, Krebs M (2011). Factors influencing the decision for Baha in unilateral deafness: the Bern benefit in single-sided deafness questionnaire. Adv Otorhinolaryngol.

[22] Hoppe U, Hocke T, Hast A (2019). Das maximale Einsilberverstehen als Prädiktor für das Sprachverstehen mit Cochleaimplantat (Maximum monosyllabic score as a predictor for cochlear implant outcome). HNO.

[23] Speck I, Ketterer MC, Arndt S (2020). Comparison of speech recognition and localization ability in single-sided deaf patients implanted with different cochlear implant electrode array designs. Otol Neurotol.

[24] Dillon MT, Buss E, Adunka MC (2013). Long-term speech perception in elderly cochlear implant users. JAMA Otolaryngol Head Neck Surg.

[25] Lenarz M, Sönmez H, Joseph G (2012). Long-term performance of cochlear implants in postlingually deafened adults. Otolaryngol Head Neck Surg.

[26] Nassiri AM, Wallerius KP, Saoji AA (2022). Impact of duration of deafness on speech perception in single-sided deafness cochlear implantation in adults. Otol Neurotol.

[27] Blamey P, Arndt P, Bergeron F (1996). Factors affecting auditory performance of postlinguistically deaf adults using cochlear implants. Audiol Neurootol.

[28] Gan J, Zhang Y, Wu J (2021). Current understanding of hearing loss in sporadic vestibular schwannomas: a systematic review. Front Oncol.

[29] Diaz RC, Cervenka B, Brodie HA (2016). Treatment of temporal bone fractures. J Neurol Surg B Skull Base.

[30] Mosnier I, Bebear J-P, Marx M, Fraysse B, Truy E, Lina-Granade G et al. Predictive factors of cochlear implant outcomes in the elderly. Audiol Neurotol 2014; 19 Suppl 1:15–20. 10.1159/000371599 10.1159/000371599 25733361

[31] Hilly O, Smith L, Hwang E, Shipp D, Symons S, Nedzelski JM et al. Depth of Cochlear Implant Array Within the Cochlea and Performance Outcome. Ann Otol Rhinol Laryngol 2016; 125(11):886–92. 10.1177/0003489416660111 10.1177/0003489416660111 27443343

[32] Gurgel RK, Duff K, Foster NL (2021). Evaluating the impact of cochlear implantation on cognitive function in older adults. Laryngoscope.

[33] Pittman CA, Ward BK, Nieman CL (2021). A review of adult-onset hearing loss: a primer for neurologists. Curr Treat Opt Neurol.

[34] Haraldstad K, Wahl A, Andenæs R (2019). A systematic review of quality of life research in medicine and health sciences. Qual Life Res.

[35] Jiam NT, Caldwell MT, Limb CJ (2017). What does music sound like for a cochlear implant user?. Otol Neurotol.

[36] Prevoteau C, Chen SY, Lalwani AK (2018). Music enjoyment with cochlear implantation. Auris Nasus Larynx.

[37] Eurich B, Klenzner T, Oehler M (2019). Impact of room acoustic parameters on speech and music perception among participants with cochlear implants. Hear Res.

[38] Körtje M, Eichenauer A, Stöver T (2021). Impact of reverberation on speech perception and sound localization accuracy in cochlear implant users with single-sided deafness. Otol Neurotol.

[39] Jacob R, Stelzig Y, Nopp P, Schleich P. Audiologische Ergebnisse mit Cochlear implant bei einseitiger Taubheit. HNO 2011; 59:453–60. 10.1007/s00106-011-2321-0 10.1007/s00106-011-2321-0 21533601

[40] Prejban DA, Hamzavi J-S, Arnoldner C, Liepins R, Honeder C, Kaider A et al. Single Sided Deaf Cochlear Implant Users in the Difficult Listening Situation: Speech Perception and Subjective Benefit. Otol Neurotol 2018; 39(9):e803-e809. 10.1097/MAO.0000000000001963 10.1097/MAO.0000000000001963 30199498

[41] Rader T, Doge J, Adel Y (2016). Place dependent stimulation rates improve pitch perception in cochlear implantees with single-sided deafness. Hear Res.

[42] Baumann U, Rader T, Helbig S (2011). Pitch matching psychometrics in electric acoustic stimulation. Ear Hear.

